# Supplementary material for: Gene Duplication and Functional Diversification of MADS-Box Genes in Malus × domestica following WGD: Implications for Fruit Type and Floral Organ Evolution
Source: Int J Mol Sci. 2024 Aug 17;25(16):8962. doi: 10.3390/ijms25168962 (PMC11354807; doi:10.3390/ijms25168962)
Supplement: Supplementary file 1 [file ijms-25-08962-s001.zip › Supplemental Figures.pdf]

Gene Duplication and Functional Diversification of MADS-Box Genes in *M.* × *domestica* Following WGD: Implications for Fruit Type and Floral Organ Evolution

College of Horticulture, China Agricultural University, Beijing, 100193, China

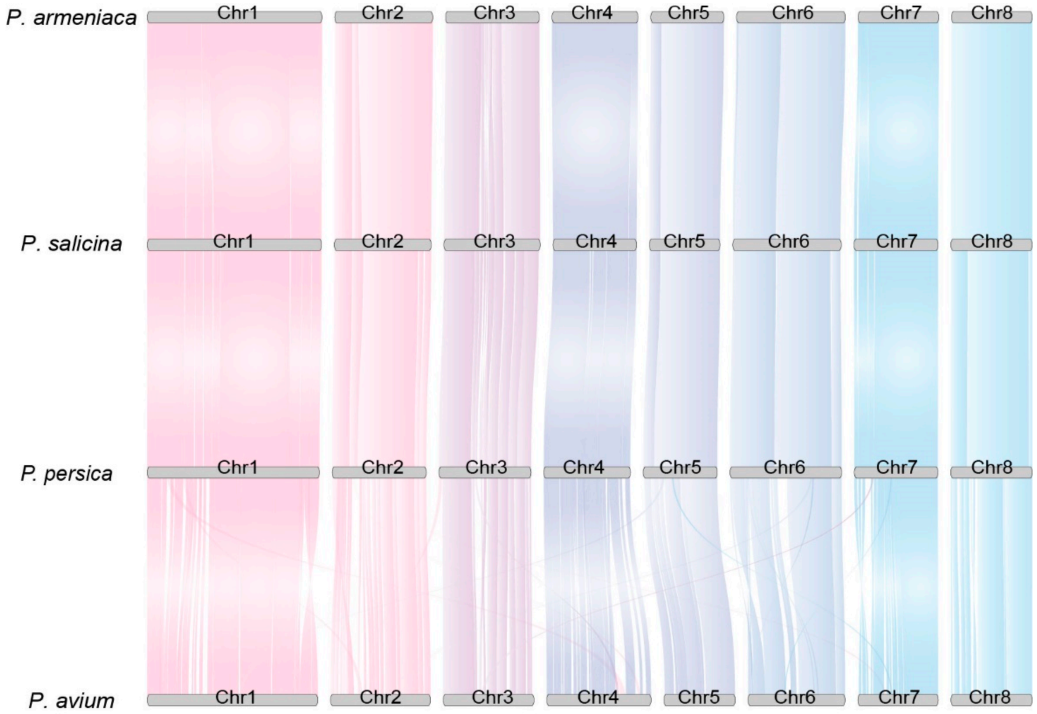

Supplemental Figure S1. Whole genome collinearity of *P. armeniaca*, *P. salicina*, *P. persica*, and *P. avium*.

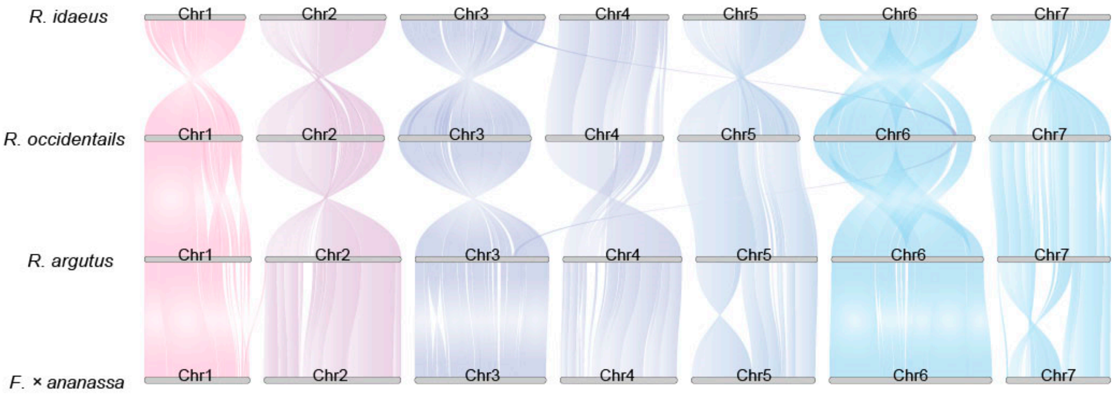

Supplemental Figure S2. Whole genome collinearity of *R. idaeus*, *R. occidentalis*, *R. argutus*, and *F.* × *ananassa*.

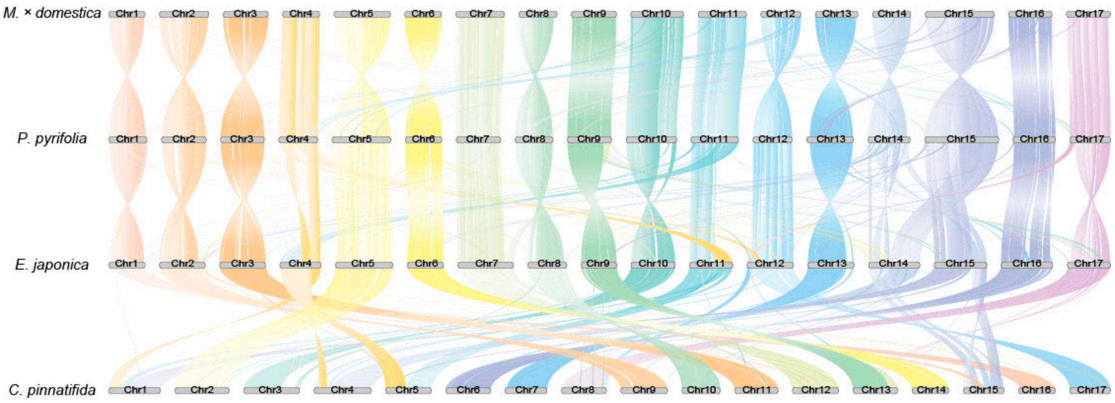

Supplemental Figure S3. Whole genome collinearity of *M.* × *domestica*, *P. pyrifolia*, *E. japonica*, and *C. pinnatifida*.

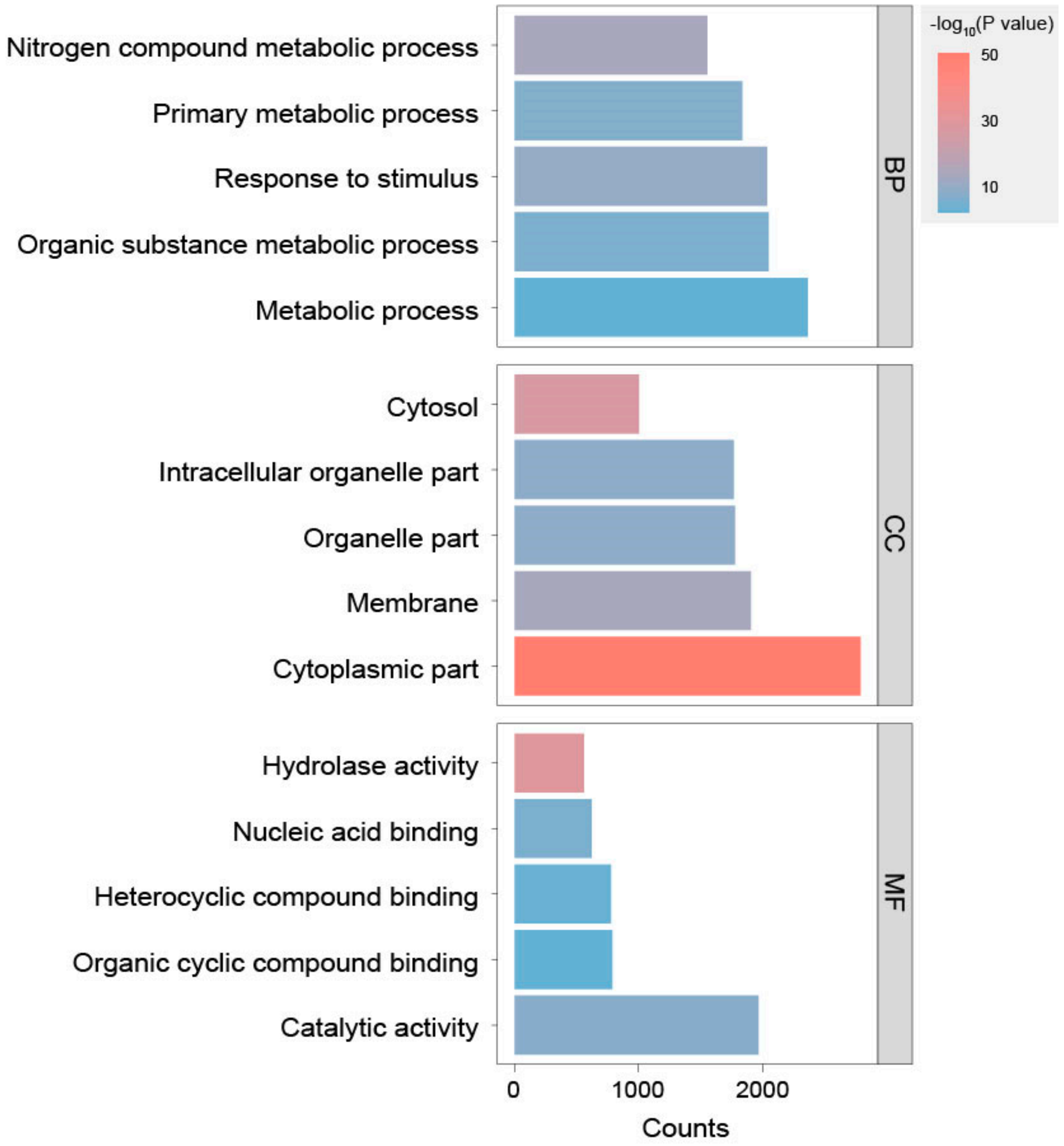

Supplemental Figure S4. Gene ontology (GO) enrichment analysis of gene preservation and expression following WGD.

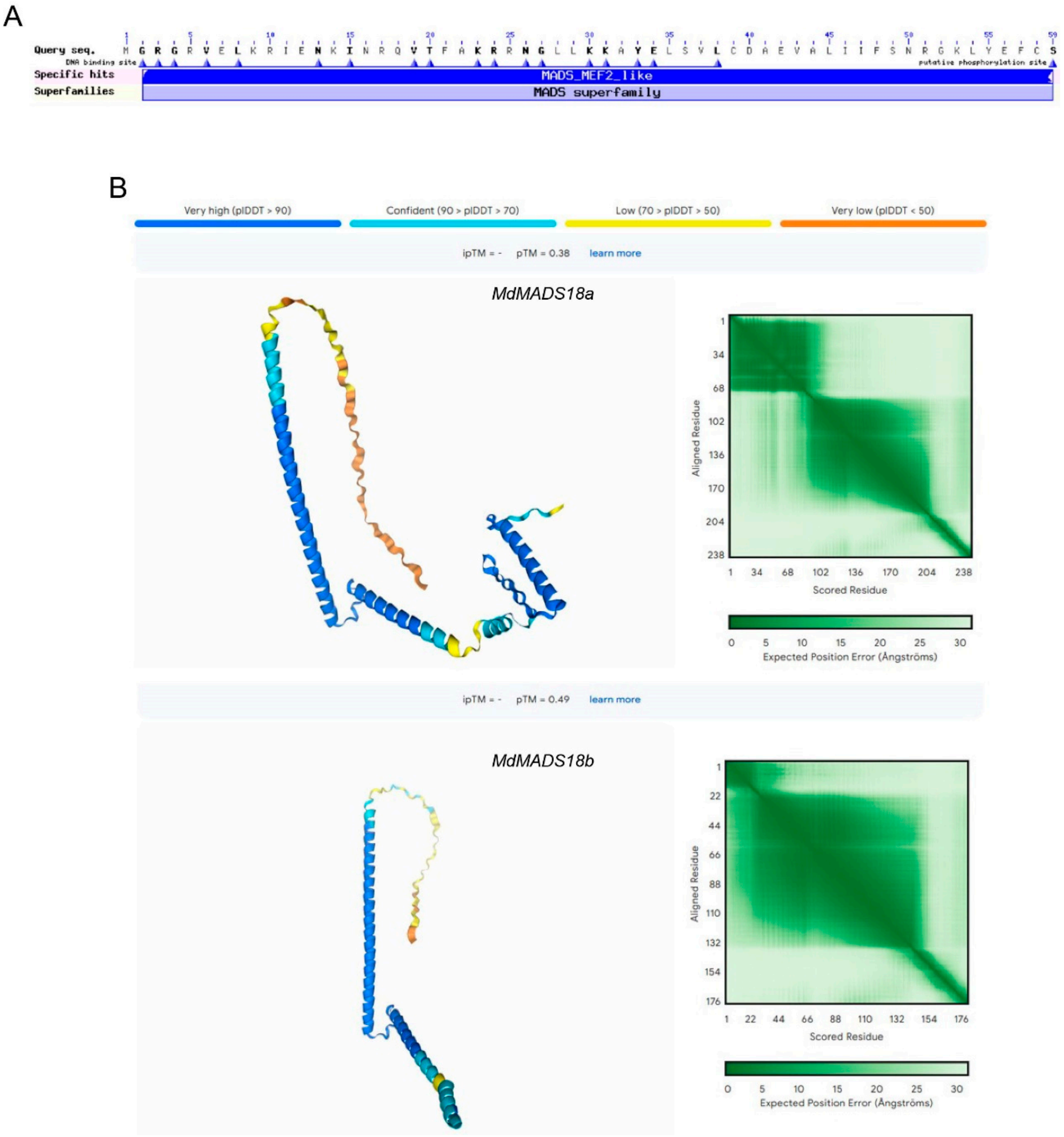

Supplemental Figure S5. Conserved domain and protein structure analysis. (**A**) The lacks 60 amino acids of *M.* × *domestica* MADS18b were submitted to the conserved domain search service of NCBI, and this polymorphic segment encodes a MEF2-like domain. (**B**) The protein sequences of *M.* × *domestica* MADS18a and MADS18b were submitted to the AlphaFold Server.

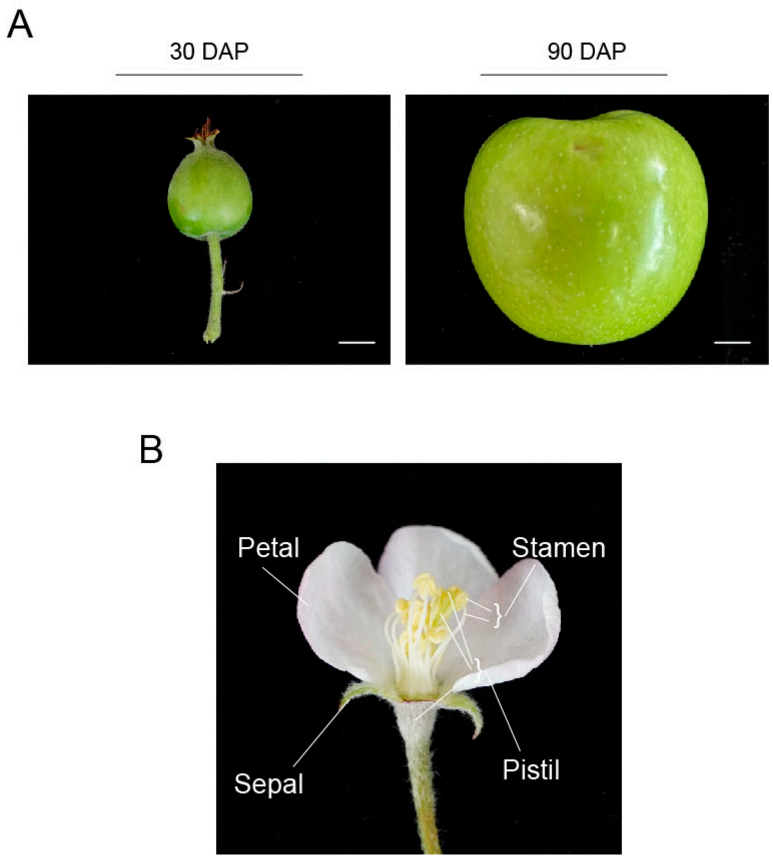

Supplemental Figure S6. The tested *M.* × *domestica* plant tissues. (**A**) Apple fruits at 30 DAP and 90 DAP, with a bar length of 1cm. (**B**) Apple flowers at the late pink bud stage include petals, sepals, pistils, and stamens. The stamens include pollen and filaments, while the pistils include the stigma, style, and ovary.

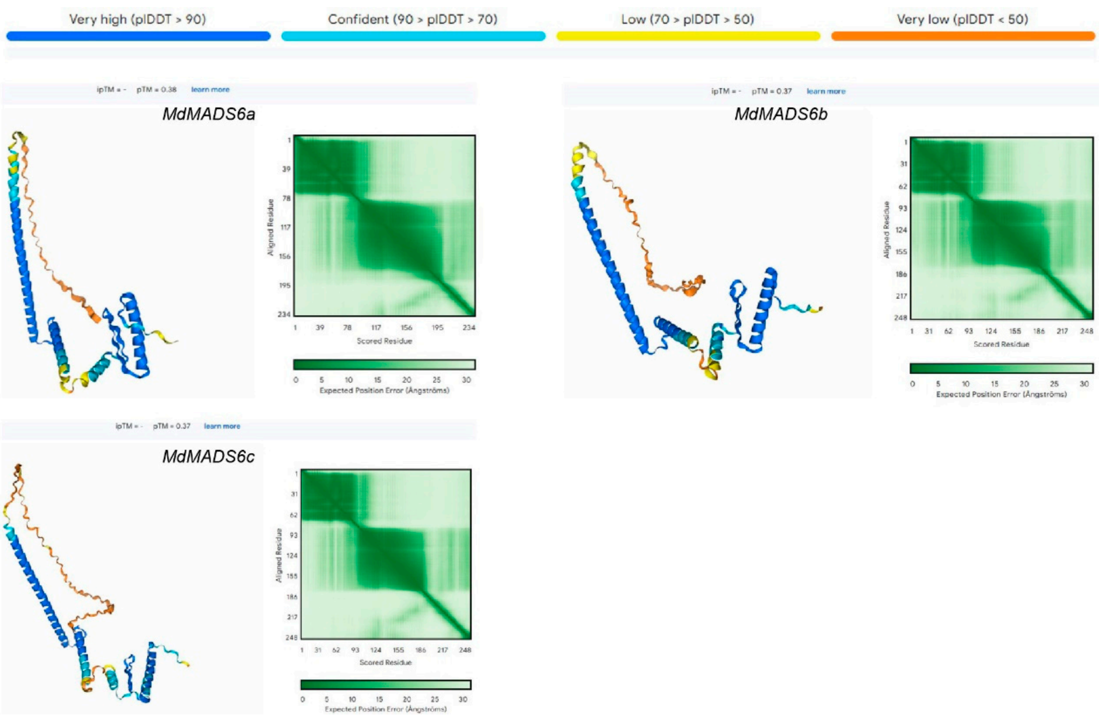

Supplemental Figure S7. The protein sequences of *M.* × *domestica* MADS6a, MADS6b and MADS6c were submitted to the AlphaFold Server generate highly accurate protein structure.
